# Effect of nalbuphine plus ropivacaine on vaginal labor in epidural analgesia

**DOI:** 10.1186/s12871-023-02209-7

**Published:** 2023-07-22

**Authors:** Guo-hua Liu, Li-wen Liu, Lian-chun Ou, Xiao-sheng Cao, Zhao Pang, Xue-jin Wen, Quan-yan He, Biao Yin

**Affiliations:** 1 Department of Anesthesia, Guangzhou Women and Children Medical Center of Liuzhou Hospital, Liuzhou City, 545000 China; 2 Department of Anesthesia, The Reproductive Hospital of Guangxi Zhuang Autonomous Region, Nanning City, 530000 China; 3 Department of Eugenic Genetics, The Reproductive Hospital of Guangxi Zhuang Autonomous Region, No.3 Longyuan Road, Qingxiu District, Nanning City, 530000 China

**Keywords:** Nalbuphine, Fentanyl, Ropivacaine, Analgesia during childbirth, Labor

## Abstract

**Background:**

Various approaches using epidural analgesia have been employed for relieving labor pain and promoting spontaneous delivery. We aimed to evaluate the effect of nalbuphine and ropivacaine versus fentanyl and ropivacaine on the duration of delivery in parturients.

**Methods:**

Clinical data of 160 full-term primiparous women who received either nalbuphine or fentanyl in combination with ropivacaine infusion for epidural labor analgesia in our hospital from December 2020 to May 2022 were retrospectively analyzed. The participants were divided into two groups based on anesthesia methods: nalbuphine group (NR group, n = 78) received 0.2 mg/mL nalbuphine combined with 0.1% ropivacaine hydrochloride for patient-controlled epidural analgesia (PCEA) and fentanyl group (FR group, n = 82) received 2 ug/mL fentanyl citrate and 0.1% ropivacaine hydrochloride for PCEA. Both groups received an epidural blockade for labor analgesia at lumbar 2–3 interspace. The duration of the first, second, and third stages of labor, the onset of analgesia, and time before delivery (T0), 15 min of analgesia (T1), 30 min of analgesia (T2), full opening of the uterine opening (T3),exerts force during childbirth(T4), heart rate (HR), blood pressure (BP), blood saturation (SpO2), visual analogue pain scale (VAS) score, Ramsay sedation score, and modified Bromage score, and 5 min were recorded at 2 h postpartum (T5). The neonatal Apgar score, neonatal behavioral neurological assessment (NBNA) score, maternal nausea, vomiting, and itchy skin were recorded.

**Results:**

Compared with the FR group, the first stage of labor duration (*p* < 0.05) and total duration of labor (*p* < 0.05) were shortened and the onset of analgesia (*p* < 0.05) was increased in the NR group. NR group had lower incidence of urinary retention than FR group (*p* < 0.05). The maternal and neonatal investigational parameters and scores had no significant difference between the two groups.

**Conclusions:**

Nalbuphine combined with ropivacaine in epidural block labor has a faster onset of analgesia and has a lower incidence of urinary retention than fentanyl combined with ropivacaine, and nalbuphine shortens the duration of the first and total stages of labor. Both nalbuphine and fentanyl can reduce pain during labor, have little effect on maternal hemodynamics, and have no significant effect on neonatal Apgar or NBNA scores.

## Background

Labor pain activates the sympathetic nervous system, which causes a significant increase in catecholamine levels in the maternal blood. This inhibits uterine contractions and prolongs the duration of labor [1]. Effective labor analgesia can reduce the maternal stress response, which is beneficial for both women and newborns [1, 2]. Currently, epidural analgesia is recognized as effective and safe [3]. Guidelines recommend the use of low-concentration local anesthetics combined with small amounts of opioids, such as fentanyl and sufentanil, to enhance analgesia while reducing the risk of movement blockade and related complications from the local anesthetics. Fentanyl belongs to the class of opioid receptor agonists, which have analgesic and sedative effects on µ1 receptors but can also cause µ2-related side effects, such as nausea, vomiting, itching, and respiratory depression. The use of fentanyl at high doses can affect maternal ventilation function, slow down the maternal heart rate, cause a drop in blood pressure, and result in pruritis or itching in the epidural space. However, nalbuphine has a partial antagonistic effect on µ receptors but can fully excite the κ receptors, which are present in high concentration in the spinal cord. This indicates that nalbuphine could effectively inhibit visceral pain and improve somatic pain [4]. In addition, nalbuphine partially inhibits µ receptors, resulting in a low incidence of pruritus. Current analgesia regimens for neuraxial delivery can extend the duration of the first, second, and total labor [5]. However, a single intermittent intravenous injection of 10 mg of nalbuphine is effective in shortening labor [6]. There is currently limited research on epidural nabuprofen combined with ropivacaine for labor analgesia.Therefore, this study aimed to evaluate the effect of epidural nalbuphine combined with ropivacaine for labor analgesia on maternal labor duration, analgesic effect, and adverse effects.

## Methods

### Study design

A total of 160 patients was included in the study. Retrospective analysis was performed with 78 parturients who received nalbuphine combined with ropivacaine and 82 parturients who received fentanyl combined with ropivacaine from December 2020 to May 2022 at our hospital. The age range was 22–37 years, gestational weeks ranged from 37 to 41 weeks, body mass index (BMI) ranged from 18 to 35 kg/m^2^, and the American Society of Anesthesiologists (ASA) grades were II. Parturients were divided into the nalbuphine group (NR group, n = 78) and fentanyl group (FR group, n = 82), based on the anesthesia method. The Medical Ethics Committee of the hospital reviewed and approved this study (NO:2020-051).Furthermore, the mother and her family provided informed consent for labor analgesia.

The inclusion criteria were antenatal assessment by an obstetrician, likelihood of vaginal delivery, mothers who voluntarily underwent vaginal trial labor and voluntary childbirth analgesia, and ASA grades II. Patients with no contraindications to epidurals were also included.

The exclusion criteria were women who underwent a cesarean section; women who were being administered sedative or analgesic drugs or had a history of alcohol or drug abuse; maternal mental illness; pregnant women with obstetric-related complications or comorbidities; those with contraindications to neuraxial anesthesia, related opioids, and local anesthetic allergies; and those who had a spontaneous transition to cesarean section in the middle of labor.

### Methods

After the two groups of women entered the delivery room, the uterine opening was dilated to 2–3 cm. An upper limb intravenous infusion of 200–300 mL of compound sodium chloride was administered to monitor heart rate (HR), blood pressure (BP), blood oxygen saturation (SpO2), and fetal heart rate. The participant was positioned on her left side, and the L2–L3 intervertebral space was selected for epidural puncture. After successful catheter placement and with negative aspiration for blood and cerebrospinal fluid, the catheter was stabilized. The patient was administered 3 mL of 1% lidocaine hydrochloride and placed in a left-inclined lying position. The patient was observed for 5 min to ensure that there were no signs of total spinal anesthesia or toxicity. The NR group was administered with an 8 mL mixture of 0.2 mg/mL nalbuphine (Hubei Yichang Renfu Pharmaceutical Co., Ltd., Sinopharm H1171102, specification 1 mL:20 mg) and 0.1% ropivacaine (AstraZeneca Pharmaceutical Co., Ltd., Switzerland, imported drug registration number: H20140763, specification 10 mL:100 mg) in a maternal epidural cavity. The FR group was administered with an 8 mL mixture of 2ug/mL fentanyl (Hubei Yichang Renfu Pharmaceutical Co., Ltd., Sinopharm H20054172, specification 2 mL:0.1 mg) and 0.1% ropivacaine (AstraZeneca Pharmaceutical, imported drug registration number: H20140763, specification 10 mL:100 mg). Participants of both the groups were connected to a patient-controlled analgesia pump with the same concentration of epidural self-controlled analgesia (PCEA). The pump delivered a continuous background dose of 8 mL/h and an autonomic dose of 8 mL/h, with a 30 min interval. Participants were instructed to use self-controlled compressions at a visual analog pain scale (VAS) score > 5 and to stop the analgesic pump after delivery.

### Observation index

The lengths of the first, second, and third stages of labor were recorded along with the HR, BP, SpO2, VAS score, Ramsay sedation score, and modified Bromage score at various time points. These time points were as follows: before delivery (T0), 15 min after analgesia administration (T1), 30 min after analgesia administration (T2), full opening of the uterus (T3), delivery (T4), and 2 h postpartum (T5). In addition, the Apgar scores of the neonates at 1 and 5 min, the neonatal behavioral neurological assessment (NBNA) score, and the incidence of adverse reactions, such as maternal nausea, vomiting, and itchy skin, were recorded.

The VAS score assessed the degree of pain on a scale of 0 (no pain) to 10 (most severe pain). A score of 1–3 indicates mild pain, 4–6 indicates moderate pain that affects sleep, 7–9 indicates severe pain and an inability to sleep, and 10 indicates the most painful sensation.

The Ramsay sedation score assesses the degree of sedation on a scale of 1–6. A score of 1 indicated that the patient was not quiet or is irritable, whereas a score of 2 indicated that the patient was quiet and cooperative. A score of 3 indicated drowsiness with the ability to follow instructions, and a score of 4 indicated a sleep state that could be awakened. A score of 5 indicates falling asleep but responding to strong stimuli, and a score of 6 indicates deep sleep from which it is difficult to wake up. A score of less than 2 indicated no sedation, 2–4 indicates satisfactory sedation, and a score greater than 4 points indicated excessive sedation.

The improved Bromage score assessed the degree of motor blockade, with a score of 0 indicating no motor block, a score of 1 indicating no hip flexion, 2 indicating an inability to flex the knee, and 3 indicating an inability to flex the ankle.

### Statistical analyses

Data were tabulated and analyzed using IBM SPSS 24.0. The measurement data, expressed as (x ± s), followed a normal distribution. Intra-group comparisons were performed using the t-test. The counting data was expressed as a percentage (%), and inter-group comparisons were performed using the *χ*^*2*^-test. Differences were considered statistically significant at *p* < 0.05.

## Results

Among the 160 women included in this study, there were no significant differences in maternal age, gestational age, and BMI index between the two groups (Table 1).

**Table 1:** Comparison of the general situation of the two groups of women (x ± s)

Group	Number of cases	Age (years)	Gestational age (weeks)	BMI(kg/m^2^)
NR Group	78	28.6 ± 2.2	39.2 ± 1.1	30.9 ± 1.2
FR Group	82	29.1 ± 2.1	39.4 ± 0.8	31.1 ± 1.1
*t*		1.471	1.320	1.100
*p*		0.143	0.189	0.273

The duration of the first and total labor in the NR group was significantly lower than that in the FR group (*p* < 0.05). There was no significant difference between the second and third stages of labor between the two groups (Table 2).

**Table 2:** Comparison of the duration of labor between the two groups (min, x ± s)

Group	Number	First stage of labor	Second stage of labor	Third stage of labor	Total stage of labor
NR Group	78	492.6 ± 51.1	66.2 ± 11.7	7.1 ± 1.2	565.9 ± 64.0
FR Group	82	587.3 ± 49.8	64.4 ± 13.5	7.3 ± 1.4	658.7 ± 64.7
*t*		11.871	0.899	0.968	9.116
*p*		< 0.001	0.370	0.335	< 0.001

Compared with T0, the maternal BP was significantly reduced in both groups at T1 (*p* < 0.05), and there were no significant differences in HR, BP, or SpO2 between the two groups at the different time points (Table 3).

**Table 3:** Comparison of HR, BP and SpO2 between the two groups(x ± s)

Indicator	Group	Number of cases	T0	T1	T2	T3	T4	T5
HR(times/ min)	NR	78	87.6 ± 11.5	84.8 ± 12.2	84.2 ± 10.9	96.5 ± 11.3	103.6 ± 17.1	88.5 ± 12.2
	FR	82	86.9 ± 10.8	84.1 ± 11.6	83.9 ± 11.7	95.7 ± 10.9	104.1 ± 16.8	87.3 ± 12.6
*t*			0.397	0.372	0.168	0.456	0.187	0.612
*p*			0.692	0.710	0.867	0.649	0.852	0.542
BP(mmHg)	NR	78	128.1 ± 8.2	115.6 ± 3.1	113.4 ± 4.2	127.5 ± 9.2	132.4 ± 8.6	118 ± 7.6
	FR	82	125.1 ± 9.6	114.8 ± 4.7	113.8 ± 5.1	126.7 ± 8.6	131.7 ± 9.2	117 ± 8.5
*t*			2.120	1.264	0.540	0.568	0.497	0.783
*p*			0.036	0.208	0.590	0.571	0.620	0.435
SpO2(%)	NR	78	97.5 ± 1.4	97.8 ± 1.2	97.6 ± 1.3	97.6 ± 1.3	97.4 ± 1.1	97.5 ± 1.2
	FR	82	97.8 ± 1.3	97.7 ± 1.3	97.7 ± 1.2	97.7 ± 1.4	97.6 ± 1.4	97.3 ± 1.3
*t*			1.405	0.505	0.506	0.468	1.001	1.010
*p*			0.162	0.614	0.614	0.641	0.318	0.314

In contrast to T0, the VAS scores of the two groups were significantly reduced at T1 (*p* < 0.05). The Ramsay sedation score was significantly high in both groups (*p* < 0.05). At T2, the Ramsay sedation score was significantly higher in the NR group than in the FR group *(p* < 0.05). At T3, the VAS score in the NR group was higher than that in the FR group (*p* < 0.05). There were no significant differences in the VAS and Ramsay scores between the two groups at T0, T1, T4, or T5 (Table 4). There was no significant difference in the Bromage scores between the two groups at any time point.

**Table 4:** Comparison of maternal VAS score, Ramsay score and Bromage score between the two groups(x ± s)

Indicator	Group	Number of cases	T0	T1	T2	T3		T4	T5
VAS	NR	78	8.0 ± 1.1	2.8 ± 1.7	2.2 ± 1.9	3.8 ± 2.5	5.6 ± 2.7		0.5 ± 1.1
	FR	82	8.0 ± 1.2	3.1 ± 1.5	2.3 ± 1.6	4.7 ± 2.8	5.6 ± 2.8		0.6 ± 1.6
*t*			0	1.185	0.361	2.141	0		0.871
*p*			1	0.238	0.719	0.034	1		0.385
Ramsay	NR	78	1.0 ± 0.3	2.1 ± 0.9	3.1 ± 0.5	2.0 ± 0.3	1.4 ± 0.6		2.2 ± 0.6
	FR	82	1.0 ± 0.2	2.1 ± 0.7	2.2 ± 0.4	2.1 ± 0.2	1.4 ± 0.5		2.1 ± 0.5
*t*			0	0	12.6	2.492	0		1.147
*p*			1	1	< 0.001	0.014	1		0.253
Bromage	NR	78	0	0	0	0	0		0
	FR	82	0	0	0	0	0		0

Similarly, there were no significant differences in the Apgar and 24-h NBNA scores between the two groups (Table 5).

**Table 5:** Comparison of 1 min, 5 min Apgar and 24 h NBNA scores of neonates in the two groups (x ± s)

Group	Number of cases	24hNBNA	Apgar	
			1 min	5 min
NR	78	38.8 ± 0.5	9.3 ± 0.6	9.8 ± 0.3
FR	82	38.7 ± 0.4	9.2 ± 0.8	9.8 ± 0.5
*t*		1.400	0.891	0
*p*		0.163	0.374	1

The incidence of adverse reactions, such as nausea, vomiting, dizziness, and pruritus, was not significantly different between the two groups (Table 6). The incidence of maternal urinary retention was significantly lower in the NR group than in the FR group (*p* < 0.05).

**Table 6:** Comparison of maternal adverse reactions in the two groups [n (%)]

Group	Number of cases	Nausea	Vomiting	Dzziness	Itching	Urinary retention
NR	78	9(12)	5(6)	12(15)	0(0)	5(6)
FR	82	7(9)	4(5)	10(12)	2(2)	17(21)
*χ* ^*2*^		0.400	0.177	0.343	1.927	6.914
*p*		0.527	0.674	0.558	0.165	0.009

## Discussion

In the present study nalbuphine combined with ropivacaine in epidural block labor showed a faster onset of analgesia, a lower incidence of urinary retention and a shortened the duration of the first and total stages of labor than that of fentanyl combined with ropivacaine. Additionally, both nalbuphine and fentanyl could reduce pain during labor with considerable effect on maternal hemodynamics without significant effect on neonatal Apgar or NBNA scores.

Childbirth is a natural physiological process that can cause severe pain to the mother due to uterine contractions and cervical dilation during labor. Childbirth analgesia is an effective means of reducing pain and reflects the degree of social civilization and humanistic care. In some developed countries, the rate of childbirth analgesia can reach approximately 80%. In recent years, the rate of childbirth analgesia in China has increased annually [7, 8]. Pain during delivery activates the sympathetic nervous system, resulting in a significant increase in catecholamine levels in maternal blood. This increase can inhibit uterine contractions, prolong the duration of labor, and lead to increased peripheral blood resistance and decreased uterine placental perfusion. Pain during labor can irritate the respiratory system, causing intermittent maternal hyperventilation and hypoventilation, which can lead to decreased oxygen saturation and hypoxia in the fetus [9]. Effective labor analgesia can relieve severe pain, improve the mood, and reduce the maternal stress response. It can also effectively prevent uterine contraction disorders, increase the uterine–placental blood flow and fetal oxygen supply, thereby improving the rate of spontaneous delivery.

Epidural analgesia provides continuous pain relief and is the safest and most effective option for labor analgesia. Ropivacaine is a long-acting amide that has both anesthetic and analgesic effects, and provides sensory and motor separation blockade. It has no effect on the uterine–placental blood flow and is widely used for epidural labor analgesia [10]. A previous study has demonstrated that using ropivacaine for epidural analgesia can extend the duration of the first, second, and overall stages of labor [11].

The results of this study showed that, at T2, the Ramsay sedation score was significantly higher in the NR group than in the FR group. However, there was no significant difference in the VAS scores between the two groups, during the first stage of labor. VAS scores in the NR group at T3 tended to increase. Durations of the first stage of labor and total labor in the NR group were shorter than those in the FR group. Additionally, the incidence of pruritus and urinary retention was lower in the NR group than in the FR group. It is speculated that the possible reason for this observation could be nalbuphine stimulating the kappa receptors, which are highly concentrated in the spinal cord, leading to a rapid and potent analgesic effect at the spinal cord level [12]. This can improve somatic and visceral pain [4]. The potent and rapid sedative effect of nalbuphine also lowered the amount of ropivacaine required in the NR group compared to that in the FR group, thereby shortening the first stage of labor and the total duration of labor. Zhiqiang [13] also found that a single intermittent intravenous injection of nalbuphine 10 mg can significantly reduce the minimum effective concentration of local anesthetics required during epidural blockade and effectively shorten the duration of labor. In the second stage of labor, maternal pain was mostly somatic, resulting in an increase in the VAS score in the NR group compared to that in the FR group. However, an increase in maternal self-control drugs reduced the pain score gap between the two groups, and there was no significant difference in VAS scores between the two groups at T4 and T5.

Besides presenting with low incidence of pruritus, low-dose nalbuphine also has a therapeutic effect on opioid-induced itching [14]. However, due to the small study population, there was no significant difference in the incidence of pruritus between the two groups. Both groups experienced urinary retention, which was related to a reduction in the maternal urge to urinate caused by the use of local anesthetics and analgesia. The incidence of urinary retention in the NR group was lower than that in the FR group, possibly due to the different receptors targeted by nalbuphine and fentanyl.

Currently, the Apgar score is a crucial tool for assessing the degree of neonatal hypoxic asphyxia. The NBNA score is a screening test used to evaluate the impact of drugs on the neonatal nervous system. It can effectively reflect the functional status of the neonatal nervous system, and a score of less than 35 indicates the inhibitory effects of sedative drugs on the neonatal nervous system. This study confirmed that both drug combination regimens had little effect on neonates and were safe for analgesia in epidural delivery, as the Apgar score and 24-h NBNA score of both the groups were within the normal range.

The limitation of our study was we did not determine the umbilical artery blood gas markers or lactate levels in newborns, and further research is necessary to fully evaluate the safety and effectiveness of this approach.

## Conclusions

The combination of nalbuphine and ropivacaine is a safe and feasible option for epidural labor analgesia. Compared with fentanyl, it can shorten the duration of labor, reduce pruritus and urinary retention, and is an effective solution for pain relief during childbirth.

## Data Availability

All data generated or analyzed during this study are included in this published article.

## Abbreviations

NR: Nalbuphine group
FR: Fentanyl group
PCEA: Patient-controlled epidural analgesia
NBNA: Neonatal Behavioral Neurological Assessment
BMI: Body mass index
ASA: American Society of Anesthesiologists
HR: Heart rate; BP: Blood pressure
SpO2: Blood oxygen saturation
VAS: Visual analog pain scale
T: Time point
